# Bayesian analysis of non-communicable diseases risk factors: a focus on the lower-educated population in Bangladesh

**DOI:** 10.1093/inthealth/ihae087

**Published:** 2024-12-09

**Authors:** Md Ismail Hossain, Moumita Datta Gupta, Tahsina Fariha Ohi, Md Mahfuzur Rahman

**Affiliations:** Department of Mathematics and Natural Sciences, BRAC University, Dhaka-1212, Bangladesh; Department of Mathematics and Natural Sciences, BRAC University, Dhaka-1212, Bangladesh; Department of Statistics, Jagannath University, Dhaka-1100, Bangladesh; Department of Mathematics and Natural Sciences, BRAC University, Dhaka-1212, Bangladesh

**Keywords:** Bangladesh, Bayesian analysis, diabetes, hypertension, lower-educated population, non-communicable diseases, risk factors

## Abstract

**Background:**

This study investigates non-communicable disease (NCD) risk factors, specifically hypertension and diabetes, among Bangladeshi adults with lower educational attainment. With an increasing global burden of NCDs, understanding the dynamics in lower-educated populations becomes crucial for targeted interventions and achieving Sustainable Development Goal 3.4–curtailing premature mortality from non-communicable diseases by one-third by 2030 through prevention and treatment.

**Methods:**

Utilizing data from the Bangladesh Demographic and Health Survey (2017–2018), a two-stage stratified sampling design identified 7287 lower-educated individuals. Bayesian logistic regression was applied for risk factor analysis.

**Results:**

The prevalence of hypertension and diabetes among lower-educated people was 31% and 9.3%, respectively. NCD prevalence (37.3%) underscored a significant health burden. Factors such as gender, age, wealth status, working status, residence and region showed significant associations with NCDs. Bayesian analysis revealed that females were 1.30 times more likely to develop NCDs, while older age groups demonstrated 4.30 times greater likelihood. Employed individuals exhibited a 43% lower risk. Wealthier households showed higher NCD likelihood and residence in the central region was associated with an 11% lower risk.

**Conclusions:**

This study highlights the high risk of developing NCDs among lower-educated females, particularly those ≥35 y of age in Bangladesh. Therefore, targeted interventions for this group are critical to reducing NCD risks, supporting national health objectives and advancing progress toward the Sustainable Development Goals.

## Introduction

The association between education level and health outcomes is well-established, with numerous studies consistently linking lower levels of education to a higher rate of both communicable and non-communicable diseases (NCDs).^1–3^ Recent research has shed light on the risk factors contributing to NCDs, emphasizing that populations with lower education levels face a significantly higher prevalence of these health threats.^4,5^ This underscores the crucial connection between education and public health, emphasizing the need for targeted interventions such as NCD prevention, anti-poverty initiatives and increased physical activity as outlined in the World Health Organization's (WHO's) Global Action Plan on Physical Activity 2018–2030 to address the growing burden of NCDs.^6^ Additionally, school-based interventions in less-educated communities can play a vital role in mitigating NCD risks and promoting healthier behaviors.^7^ Globally, NCDs, encompassing cardiovascular or heart diseases, hypertension, diabetes, cancer and chronic respiratory diseases, constitute a primary cause of mortality. Every year, 41 million deaths worldwide (74% of the total fatalities) are linked to NCDs, while 77% of the fatalities that take place in lower- and middle-income countries (LMICs) are caused by NCDs according to the Noncommunicable Diseases Progress Monitor 2022 from the WHO.^8^ In fact, according to the 2023 WHO report, about 86% of premature mortality from NCDs occurs in LMICs.^9^ Meanwhile, the NCD Alliance's 2019 survey projects that the annual death toll from NCDs will increase to nearly 52 million, while deaths from infectious diseases are expected to decrease.^10^ This epidemiological trajectory accentuates the transition of NCDs from emerging concerns to an alarming status, reminiscent of an epidemic, particularly in developing nations.^11–13^

The 2023 WHO report identifies cardiovascular diseases as the major cause of most of the deaths caused by NCDs (around 17.9 million individuals per year, constituting 31% of all deaths worldwide and 44% of all deaths from NCDs).^14^ It found that elevated systolic blood pressure (SBP) is one of the leading treatable exposures globally, linked to a significant number of premature cardiovascular deaths.^12,13,15^ Also, hypertension is globally acknowledged as one of the preventable major risk factors for cardiovascular diseases and their associated deaths.^16^ According to the WHO, hypertension is another leading cause of early mortality across the world, which is mostly common among adults ages 30–79 y residing LMICs. Nearly 46% of these individuals are unaware of the fact that they have hypertension, thus only 42% of hypertensive adults are diagnosed and treated.^17^ Similarly, regarding diabetes mellitus, a substantial risk factor for kidney and cardiovascular diseases, two million individuals succumb to its impact annually.^18^ Although this figure is comparatively lower than for other NCDs, diabetes mellitus remains a burning issue, particularly in LMICs, where its prevalence increased by 13% from 2000 to 2019.^15^ Considering the extensive literature reviewed, it is evident that the exploration of NCDs remains a vital undertaking. This is especially true for LMICs, where understanding and addressing NCDs play a pivotal role in working towards the accomplishment of Sustainable Development Goal (SDG) 3.4.

A noticeable increase in health challenges is particularly evident in South Asian countries. In Southeast Asia, a staggering nine million deaths are linked to NCDs, making up 62% of all recorded deaths.^19^ In the year 2021, NCDs were responsible for about two-thirds of all deaths that occurred within this region, with half of these deaths taking place among individuals 30–69 y of age.^20^ Similar to many other LMICs and other South Asian countries, Bangladesh is also encountering a rapid escalation in NCDs as a result of changes in sociodemographic factors and epidemiological shifts.^21^ Bangladesh experiences approximately 572 600 deaths annually due NCDs, which accounts for 67% of all deaths, with 22% of these deaths being potentially considered as premature.^22^ Bangladesh shoulders a significant burden of diabetes, with an estimated 8.4 million adults diagnosed with the disease.^23^ A recent study in Bangladesh reports that diabetes rates among people ≥35 y of age increased from about 11% in 2011 to 14% in 2018,^24^ surpassing India's rate (around 9%)^25^ but remaining lower than Malaysia's (approximately 19%).^26^ Also, hypertension is the primary preventable contributor of numerous health issues, causing a significant economic burden for the people of Bangladesh.^27,28^ It is estimated that hypertension is responsible 15–20% of all NCD-related deaths in this Bangladesh.^29^

Multiple studies indicate an association between lower educational attainment and elevated rates of both hypertension and diabetes among respondents. For example, in the past, research conducted in Brazil found that people with less education had more diabetes, high BP and heart issues.^1^ Research in China revealed that people with less education often struggle to manage hypertension properly.^5^ A follow-up study conducted by Sun et al.^4^ in China between 2014 and 2016 found that individuals with a primary-level education or below faced a higher risk of being recently diagnosed with hypertension and encountered difficulties in controlling BP when compared with their counterparts with a secondary education or above. Recognizing this disparity, it is imperative to investigate and identify the specific determinants of NCDs among Bangladeshi people, particularly within the context of individuals with lower education levels.

In light of the existing research gap, this study aims to uncover the influential factors related to NCDs among the lower-educated population in Bangladesh. Earlier Bangladeshi researchers predominantly utilized a multilevel logistic regression approach, employing the maximum likelihood estimation procedure,^30^ to determine the contributing factors responsible for NCDs by estimating unknown parameters. Compared with classical inference, Bayesian inference not only gives more accurate estimates but also incorporates prior information, capturing more uncertainty. It provides a natural way to quantify uncertainty in parameter estimates through the posterior distribution, which is more intuitive and interpretable than frequentist confidence intervals.^31^ Consequently, this study employs a Bayesian logistic regression approach to achieve its objectives. Since there is no historical data with similar characteristics to develop an informative prior, a non-informative prior is used to allow the data to predominantly drive the inference.

## Methods

### Data sources

This research draws on the findings from the Bangladesh Demographic and Health Survey (BDHS), 2017–2018, a nationally representative dataset widely acknowledged for its scope and reliability. Spearheaded by the National Institute of Population Research and Training (NIPORT), this extensive survey was made possible through generous funding provided by the United States Agency for International Development (USAID). The BDHS dataset, renowned for its accessibility and comprehensiveness, is openly accessible to researchers and policymakers.

### Sample design

**Figure 1.**
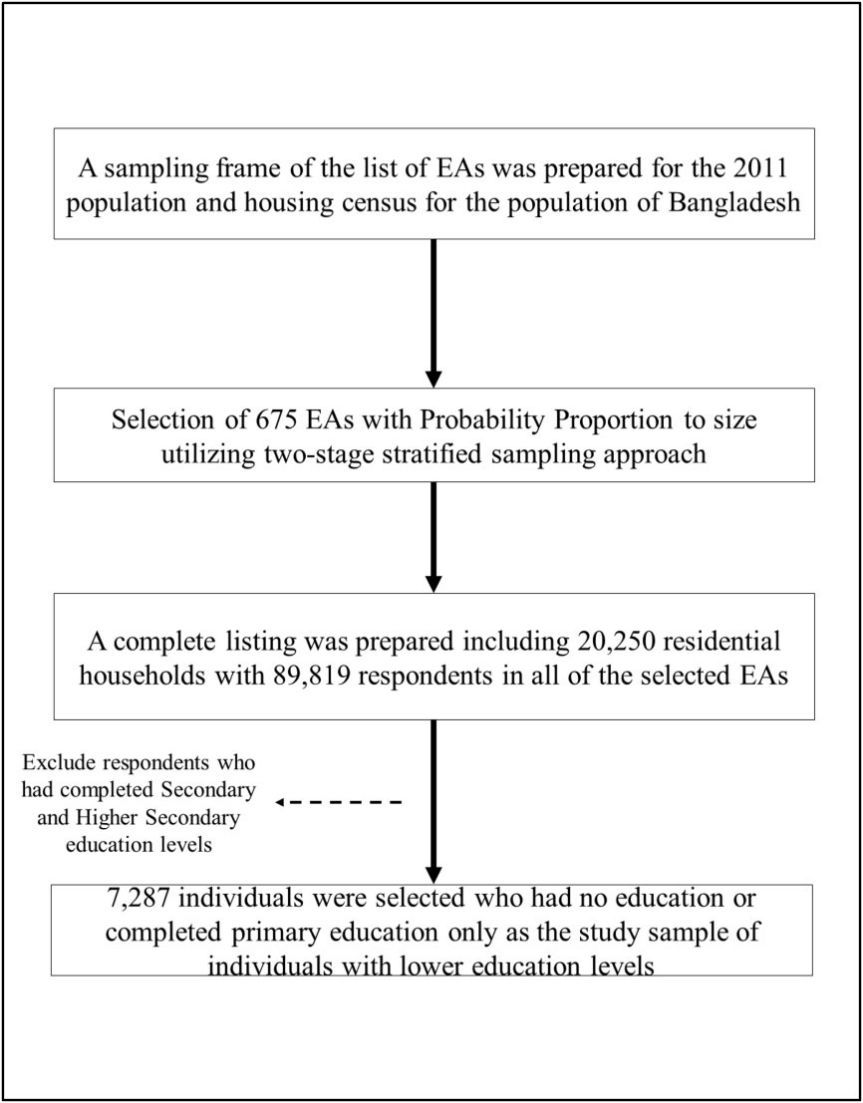
Study population and sample selection procedure for this study.

The BDHS 2017–2018 utilized a two-stage stratified sampling approach by selecting 675 enumeration areas (EAs) and then chose 30 households from each EA. The survey covered 20 250 households with 89 819 respondents. To specifically study the lower-educated population, the study identified individuals with no education or who had completed primary education; respondents with secondary or higher education levels were excluded from the sample. Weighting of a nationally representative dataset is important to ensure an accurate representation of the nation's population structure. By utilizing suitable weighting factors provided in the BDHS, the study finally included 7287 individuals with lower education levels. The flow chart in Figure 1 provides a brief idea about how the individuals were selected in the study.

### Dependent variable

The study's dependent variable focused on the NCD status among lower-educated respondents, specifically evaluating their hypertension status and diabetes status. According to American Heart Association guidelines, a respondent was considered to have hypertension if their SBP was ≥140 mmHg or their diastolic blood pressure (DBP) was ≥90 mmHg, or if they were taking antihypertensive medication.^32^ For diabetes assessment, the study adhered to the WHO guidelines, identifying individuals with diabetes if their fasting plasma glucose level was ≥7.0 mmol/l.^33^ As NCD denotes the presence of either hypertension or diabetes, this study redefines NCD in the following way: presence of hypertension or diabetes as 1, otherwise 0.

### Explanatory variables

Based on a review of the literature, this study incorporated various social, demographic and economic factors as explanatory variables. These factors encompassed gender,^34^ age of the respondent in years),^35^ marital status,^35^ wealth status,^36^ place of residence,^37^ respondent's occupational status^34^ and geographical region.^37^ Instead of using five categories of wealth status, this study grouped them into three categories by combining the first two categories (poorest and poorer) into the poor category, middle was labelled as middle and the last two categories (rich and richest) were combined into the rich category. Additionally, the geographical regions are categorized into four groups: Rajshahi and Rangpur as the northern region, Chattogram and Sylhet as the eastern region, Dhaka and Mymensingh as the central region and Barisal and Khulna as the southern region.

### Statistical analysis

The overall analytical section was divided into three parts. First, a simple descriptive analysis was employed to illustrate the actual proportion of the variables considered in the study. Second, the test of independence was used as a bivariate analysis that examined the relationship between NCD status in lower-educated individuals and various independent variables. To measure this association, the χ^2^ test statistic was applied. Finally, in the multivariable analysis, the Bayesian logistic regression method was applied to determine the significant exposures or risk factors that were associated with NCD status among the lower-educated population in Bangladesh. The Bayesian logistic regression approach is performed utilizing Bayes’ theorem:


\begin{eqnarray*}
f( {\gamma {\mathrm{|}}{{D}_i},{{E}_{ip}}} ) = f( {{{D}_i}{\mathrm{|}}\gamma } ) \times f( \gamma ),\end{eqnarray*}

where

${{D}_i} = {\mathrm{binary\ dependent\ variable\ for\ the\ }}{{i}^{{\mathrm{th}}}}{\mathrm{\ observation}}$ ,

$f( {\gamma {\mathrm{|}}{{D}_i},{{E}_{ip}}} ) = {\mathrm{posterior\ distribution\ of\ }}\gamma $ ,

$f( {{{D}_i}{\mathrm{|}}\gamma } ) = {\mathrm{likelihood\ function}}$ ,

$f( \gamma ) = {\mathrm{prior\ distribution\ }}\gamma $ .

In Bayesian inference, two types of prior information are used: informative and flat/non-informative. This study employed a flat/non-informative prior, specifically the common non-informative prior distribution for Bayesian logistic regression parameters, expressed as


\begin{eqnarray*}{{\gamma }_j}\sim N\big( {{{\mu }_j},\sigma _j^2} \big).\end{eqnarray*}

For the specification of the non-informative prior, the most popular choice is ${{\mu }_j} = 0$ and $\sigma _j^2 = {{10}^6}$ (large enough).^31^ This study utilized the Metropolis–Hastings algorithm in Bayesian Markov chain Monte Carlo (MCMC) approximation to compute the estimated marginal posterior distributions for unknown parameters. The expected values of these distributions were used as the estimated regression coefficients in the Bayesian logistic approach, providing credible intervals instead of classic confidence intervals. Monte Carlo error, influencing parameter estimates, was addressed through an extensive run, including an initial convergence phase of 500 iterations and a long run of 150 000 iterations per chain with thinning of every 99th element. Convergence was assessed using four Markov chains and trace plots, ensuring the reliability of the findings.

### Analytical software

The complete analysis procedure was carried out in Stata 16 (StataCorp, College Station, TX, USA). Bayesian logistic regression was implemented using the Stata package bayesmh.

## Results

Our study cohort comprises individuals with no formal education or those who have completed primary education. Table 1 presents the background characteristics of the study sample. Within our study cohort, a gender-diverse population was observed, with 58% of participants being female. The majority of respondents (71.8%) were in the age group of ≥35 y, indicating substantial participation of middle-aged and elderly individuals. Geographically, most participants resided in rural areas (77%) and in the central region of Bangladesh (31.9%). Furthermore, a significant portion of the respondents were actively engaged in the workforce, with 62.9% of them being employed during the survey time.

**Table 1.** Prevalence of hypertension, diabetes and NCDs among the lower-educated population.

				NCD	
Variables	n(%)	Hypertension, %	Diabetes, %	Yes, %	χ^2^ value (p-value)
Gender					
Male	3058 (42.0)	24.7	8.8	31.9	62.24 (<0.001)
Female	4229 (58.0)	35.6	9.7	41.2	
Age (years)					
18–35	2058 (28.2)	12.2	4.8	16.8	492.69 (<0.001)
≥35	5229 (71.8)	38.4	11.1	45.4	
Wealth status					
Poor	3723 (51.1)	27.7	6.0	32.4	94.75 (<0.001)
Middle	1516 (20.8)	32.8	8.7	38.6	
Rich	2048 (28.1)	35.8	16.1	45.6	
Working status					
Not working	2700 (37.1)	40.2	12.7	47.4	178.17 (<0.001)
Working	4587 (62.9)	25.6	7.4	31.4	
Residence					
Urban	1677 (23.0)	31.7	11.7	39.6	4.51 (0.03)
Rural	5610 (77.0)	30.8	8.6	36.6	
Region					
Northern	2011 (27.6)	32.8	6.2	37.0	16.19 (0.001)
Eastern	1671 (22.9)	32.7	10.7	39.1	
Central	2328 (31.9)	25.9	11.5	34.3	
Southern	1277 (17.5)	35.2	8.8	40.7	

Table 1 also presents the prevalence of diabetes and hypertension among the lower-educated population. In our study involving 7287 individuals with lower education, we found that 31% of participants had hypertension. A higher prevalence was observed among females (35.6%). Additionally, individuals ≥35 y of age exhibited a higher prevalence (38.4%), indicating the age-associated vulnerability to hypertension in our studied population. Notably, participants from affluent households, classified as the rich wealth status group, showed a substantial prevalence of hypertension (35.8%). Moreover, a considerable proportion of non-working individuals (40.2%) also suffered from hypertension. Diabetes, another notable NCD, impacted 9.3% of the participants. Analysis revealed a slightly higher prevalence among females (9.7%) and individuals ≥35 y of age (11.1%). The affluent group displayed a noticeably higher prevalence of diabetes (16.1%) compared with the poor and middle-class groups (6.0% and 8.7% respectively), emphasizing the potential influence of socio-economic factors on the occurrence of this disease.

The prevalence of NCDs and their association with proximate determinants are displayed in Table 1. A substantial percentage of our study participants were found to experience NCDs, indicating a considerable health burden for Bangladeshi people. After conducting further analysis, the results showed a heightened prevalence of NCDs among females (41.2%) and adults ≥35 y of age (45.4%) in their susceptibility to both diabetes and hypertension. Also, the results show that the wealthier group exhibited a concerning prevalence of NCDs (45.6%), emphasizing the significant role of socio-economic factors.

**Figure 2.**
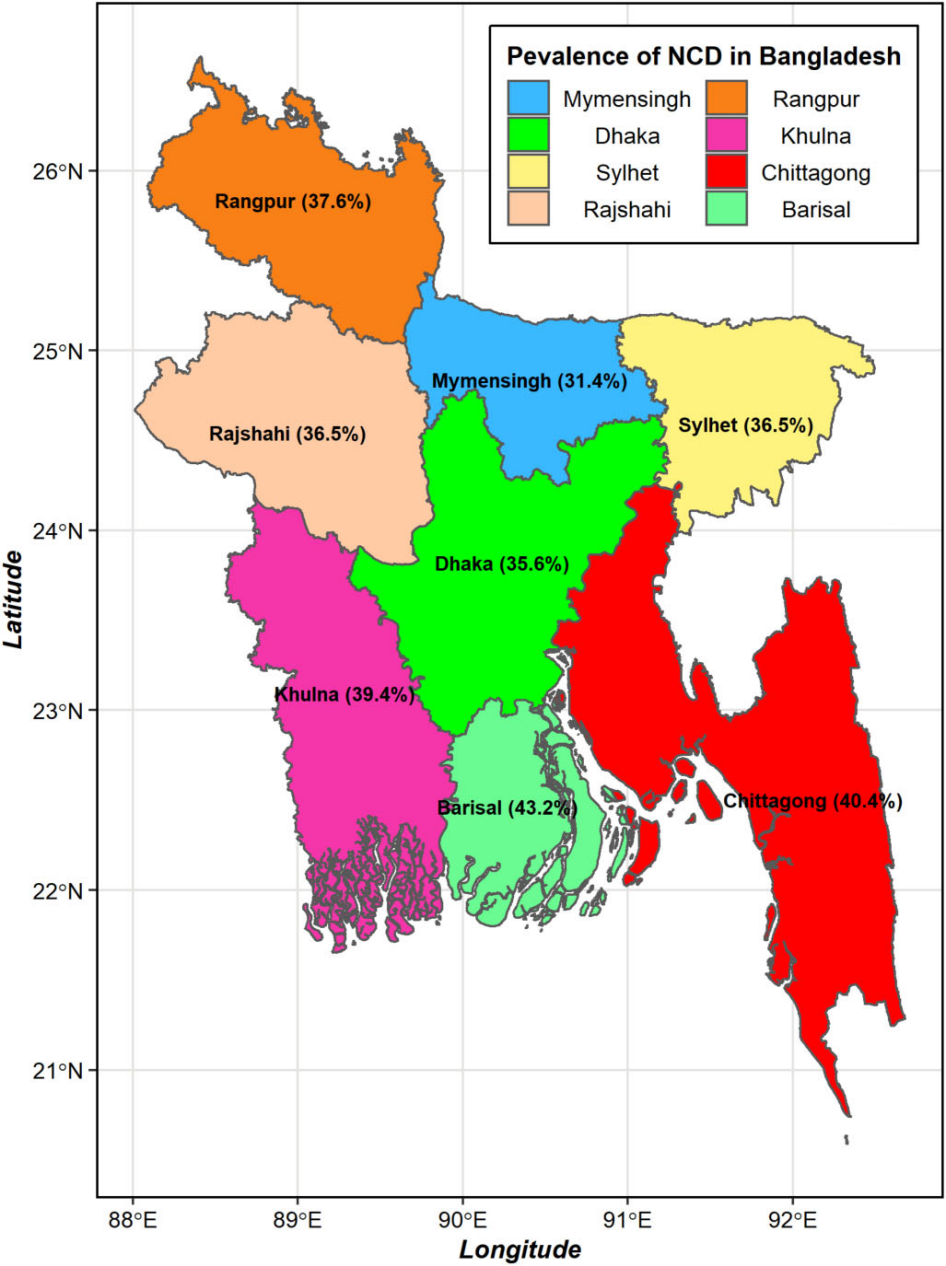
Prevalence of NCDs by division of Bangladesh.

Moreover, investigating the relationships among various potential determinants, our analysis revealed some intriguing patterns. The findings of the study unveiled evidence of gender disparities, with a higher prevalence of NCDs in females than males (χ^2^ = 62.24, p<0.001). Additionally, age played a crucial factor in the prevalence of NCDs, with a significantly sharp increase among study participants ≥35 y of age (χ^2^ = 492.69, p<0.001)$.$ Wealth status showed significant differences, indicating a higher NCD burden among affluent households (χ^2^ = 94.75, p<0.001). Employment status also emerged as a contributing factor, with non-working individuals exhibiting a heightened prevalence of NCDs (χ^2^ = 178.17, p<0.001). Geographical disparities were notable, particularly in the southern region, where a considerable prevalence of NCDs was observed (χ^2^ = 16.19, p<0.001). Figure 2 displays the regional variations in the prevalence of NCDs. The Barisal division has the highest prevalence (43.2%), followed by Chattogram (40.4%), while Mymensingh has a lower prevalence (31.4%).

This study employed a Bayesian logistic regression method to determine factors related to NCDs among individuals with lower education levels in Bangladesh. The adjusted odds ratios (ORs) with 95% confidence intervals (CIs) are presented in Table 2. Parameter estimation for this model utilized the MCMC through the Metropolis–Hastings algorithm, applying four Markov chains. The MCMC convergence at 150 000 iterations (per chain) proceeds an initial burn-in phase of 500 samples for stabilization and retention of each 99th element in the chain, as evidenced by the trace plot.

**Table 2.** Adjusted ORs for risk factors associated with NCDs among the lower-educated population in Bangladesh.

Variables	Adjusted OR	95% CI
Gender		
Male (ref.)		
Female	1.30	1.15 to 1.46
Age (years)		
18–35 (ref.)		
≥35	4.30	3.76 to 4.91
Wealth Status		
Poor (ref.)		
Middle	1.21	1.06 to 1.39
Rich	1.64	1.44 to 1.87
Working status		
Not working (ref.)		
Working	0.57	0.51 to 0.64
Residence		
Urban (ref.)		
Rural	0.96	0.85 to 1.08
Region		
Northern (ref.)		
Eastern	0.92	0.79 to 1.06
Central	0.81	0.70 to 0.93
Southern	1.00	0.86 to 1.16

Based on Bayesian inference, this study uncovered a significant relationship between respondent's gender and NCDs. Females demonstrated a 30% higher likelihood of NCDs compared with males (OR 1.30 [95% CI 1.15 to 1.46)]. Additionally, respondents ≥35 y of age were 4.30 times more susceptible to NCDs than those 18–35 y of age (OR 4.30 [95% CI 3.76 to 4.91)].

Regarding respondent working status, it significantly impacted NCDs among the lower-educated population in Bangladesh. Employed respondents had a 43% lower risk of NCDs than unemployed ones (OR 0.57 [95% CI 0.51 to 0.64]). Notably, individuals from middle-class and affluent families were 1.21 and 1.64 times more likely, respectively, to have NCDs than those from poor families. This underscores a significant positive relationship between the economic status of households and NCDs among the lower-educated population in Bangladesh.

Analysing the results in Table 2, respondents residing in the central region of Bangladesh (Dhaka and Mymensingh) exhibited an 11% lower likelihood of NCDs compared with those in the northern region (OR 0.81 [95% CI 0.70 to 0.93]).

**Figure 3.**
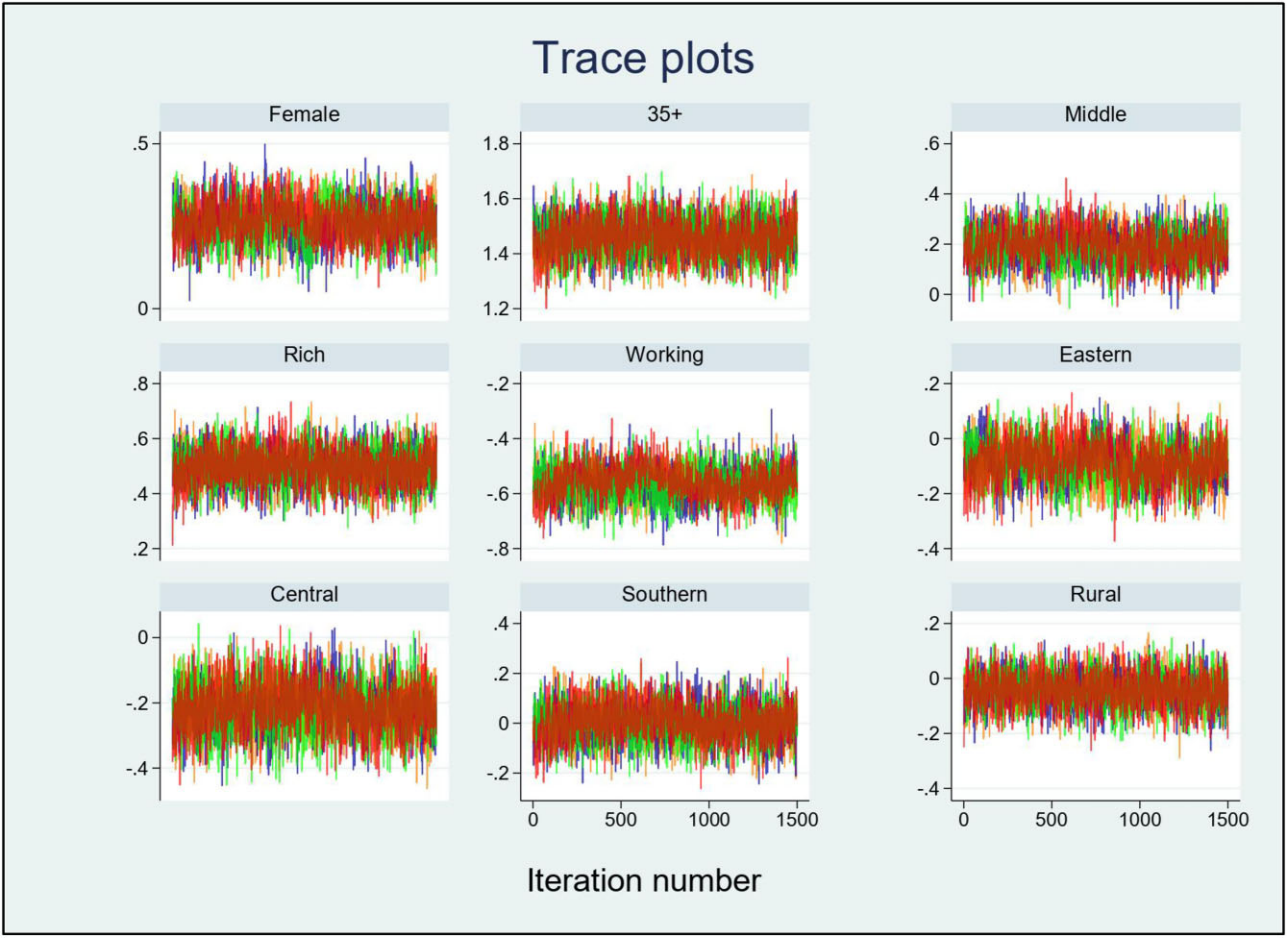
Trace plots of model parameters of Bayesian logistic regression.

Analysing trace plots is important for assessing MCMC algorithm convergence. Figure 3 displays posterior distributions for model coefficients with flat priors. Trace plots show sample overlap, confirming successful Bayesian inference. The Gelman–Rubin diagnostic confirms convergence when R_c_<1.1 for all parameters, providing confidence that convergence has been achieved.

## Discussion

This article presents the current evidence base concerning NCD risk factors among Bangladeshi adults with lower educational attainment. This study represents the initial attempt to determine NCD risk factors within a specific population by utilizing Bayesian logistic regression analysis. The findings reveal that nearly 3 of 10 adults with no or primary education have hypertension and 1 in 5 adults is diagnosed with diabetes. Older adults (≥35 y) with low education levels show a high prevalence of NCDs (45.4%), indicating a positive association. These results align with a previous study among Bangladeshi adults in slum areas with no formal or primary education.^38^ Our results regarding the elevated occurrence of NCDs among less-educated adults in Bangladesh closely aligns with prior research findings, which also indicated heightened rates of NCDs^39,40^ such as diabetes^41–43^ and hypertension.^44^ This may be because individuals with lower educational attainment often have a lower socio-economic status. Consequently, lower levels of education may serve as a mediating factor influencing both socio-economic status and health behaviours among adults within that education bracket. Furthermore, lower levels of education are often associated with poor income and insecure employment situations, and these factors have been consistently linked to an increased likelihood of NCD occurrence, as evidenced by prior research.^40,45^ Moreover, individuals with lower education levels may be more prone to unhealthy behaviours like smoking, excessive alcohol consumption, physical inactivity and poor dietary choices, all of which heighten their risk for NCDs.^46^

On a global scale, it was reported that the prevalence of NCDs was significantly higher in women compared with men in 2016,^47^ and Bangladesh is experiencing a similar pattern.^48^ Reports from different years (2017 and 2021) highlight the increasing burden of NCDs (approximately double) among women of reproductive age in LMICs.^49,50^ The situation remains the same for Bangladesh.^51^ In our study, notable gender disparities were observed in the prevalence rates of diabetes and hypertension among less-educated individuals in Bangladesh, with women exhibiting a higher prevalence of experiencing these conditions. Our findings aligned with a preceding study executed in Bangladesh, which also reported a comparatively higher likelihood of experiencing multiple risk factors of NCD among women with no education compared with men.^35^ Similar results were also found in a previous study for diabetes, hypertension and other NCDs that was conducted globally.^52^

The findings of our study show an increasing trend of prevalence of hypertension and diabetes as age and wealth increase among less-educated adults in Bangladesh, similar to previous nationwide studies for Bangladesh and elsewhere.^35,53,54^ These studies revealed a clear disparity in the prevalence of diabetes and hypertension among the illiterate population. Our findings also support this trend, showing that employed adults with low education status are less likely to be afflicted with diabetes and hypertension compared with their non-working counterparts with similar educational backgrounds. Another previous study yielded similar results, with the authors suggesting that individuals with lower educational attainment may engage in more physical activities compared with individuals with higher levels of education.^55^ Similar to other nationwide studies, our results indicate diabetes and hypertension prevalences differ depending on wealth index and geographical area.^53,56^

In our study, both diabetes and hypertension showed a significant increase corresponding to the wealth index. The strong positive relationship with the socio-economic index indicates a reduction in physical activity resulting from their immobile lifestyle. Consistent with prior nationwide studies in Bangladesh, we observed higher prevalence rates of diabetes and hypertension among adults with low levels of education residing in the southern and eastern regions of the country.^53,57^

The occurrence of NCDs may be attributed to factors like poverty, malnutrition and patterns of salt consumption.^58^ This disparity can be attributed to factors like geographic distribution, limited health infrastructure and insufficient human resources, which may restrict access to knowledge and preventive care for disadvantaged populations.^59,60^ Consequently, this could influence the distribution of factors responsible for NCDs. The results of the study also show that adults with lower educational attainment living in rural areas are more prone to NCDs compared with those with similar education levels in urban areas. Earlier studies revealed that women living in rural regions are more likely to be underweight due to insufficient access to nutritious food.^37^ Inadequate nutrition can result in significant metabolic alterations, increasing the susceptibility to heart disease, diabetes and hypertension among women and potentially affecting their future generations as well.^61^

Our study highlighted that lower education serves as a plausible covariate for NCDs. This compelled us to focus our study on less-educated Bangladeshi adults, a factor that was also noted in a previous research study,^53^ although it was not found as a significant potential covariate for causing NCDs in other studies of Bangladesh.^57,62^

Despite Bangladesh's achievement of Millennium Development Goal 5,^63^ this study found that adults with little or no education continue to face vulnerability regarding NCDs. These results suggest a growing burden of NCDs among residents ≥35 y of age with lower education in Bangladesh, potentially impeding progress towards SDG attainment. Therefore, our study suggests implementing awareness programs and other healthcare initiatives at the root level targeting these diseases, enabling early interventions to alleviate the increasing burden on our vulnerable healthcare infrastructure.

## Strengths and Limitations

The significant advantage of our study lies in the employment of a population-based, nationwide representative dataset. By encompassing both rural and urban areas throughout all administrative sectors, the BDHS ensured the generalizability of our findings to the entire country. In contrast to many previous studies, our study utilizes the Bayesian logistic regression model, which improves the accuracy and credibility of the estimation process with more precise CIs of the estimated parameters. The other strength of this article is that we explain the relationship between education level and sociodemographic and economic factors contributing to NCD prevalence, especially for adults with lower levels of education. Still, the study possesses a few limitations. Since we utilized data from a cross-sectional survey, which is secondary source data, our analysis was limited to exploring the relationships among the available response and explanatory variables in the dataset. The study could only incorporate NCDs like diabetes and hypertension, but was unable to include NCDs such as chronic respiratory diseases, cancer and heart diseases because data were unavailable. Moreover, due to insufficient information, the study could not distinguish between respondents with type 1 and type 2 diabetes. Additionally, other than the socio-economic factors, no other information was available regarding several crucial clinical biomarkers such as blood lipid profile, haemoglobin A1c and serum creatinine, along with important behavioural factors like alcohol consumption and duration of sleep. Similarly, dietary factors such as the type and quantity of food consumed, as well as physical activity levels, health behaviour and accessibility to healthcare services were not included despite being significant influential factors for NCDs. Additionally, no mediating factors like obesity, previous disease history, health conditions, lifestyles or other risk factors were considered during the study since the BDHS did not record such information.

## Conclusions

This study highlights the educational disparities in the occurrence of NCDs in Bangladesh, with a specific focus on those ≥35 y of age with lower educational attainment, in whom the prevalence rate of such conditions is particularly alarming. The prevalence of NCDs is higher among less-educated non-working adult women who belong to the affluent socio-economic groups and those who are rural residents, particularly in the eastern and southern geographic regions of Bangladesh. The findings suggest that targeted policy interventions focused on less-educated adults are essential to reduce NCD risk and meet national and SDG health goals. Delaying action may lead to worsening health outcomes and hinder progress toward sustainable development targets related to NCDs.

## Data Availability

The data underlying this study are third party and belong to the DHS Program. This study used secondary data from the BDHS 2017–2018. The dataset can be obtained from the DHS website (https://dhsprogram.com/data/available-datasets.cfm) and is publicly available upon reasonable request.
